# Interactive Education on Sleep Hygiene with a Social Robot at a Pediatric Oncology Outpatient Clinic: Feasibility, Experiences, and Preliminary Effectiveness

**DOI:** 10.3390/cancers14153792

**Published:** 2022-08-04

**Authors:** Kelly L. A. van Bindsbergen, Hinke van der Hoek, Marloes van Gorp, Mike E. U. Ligthart, Koen V. Hindriks, Mark A. Neerincx, Tanja Alderliesten, Peter A. N. Bosman, Johannes H. M. Merks, Martha A. Grootenhuis, Raphaële R. L. van Litsenburg

**Affiliations:** 1 Princess Máxima Center for Pediatric Oncology, 3584 CS Utrecht, The Netherlands; 2 Emma Children’s Hospital, Amsterdam University Medical Centers, 1105 AZ Amsterdam, The Netherlands; 3 Department of Computer Science, Vrije Universiteit Amsterdam, 1081 HV Amsterdam, The Netherlands; 4 TNO, Organization for Applied Scientific Research, 3769 DE Soesterberg, The Netherlands; 5 Department of Intelligent Systems, Delft University of Technology, 2628 XE Delft, The Netherlands; 6 Department of Radiation Oncology, Leiden University Medical Center, 2300 RC Leiden, The Netherlands; 7 Centrum Wiskunde & Informatica, 1098 XG Amsterdam, The Netherlands

**Keywords:** sleep hygiene, interactive education, social robots, pediatric oncology, psycho-oncology, innovation, outpatient clinic

## Abstract

**Simple Summary:**

Sleep problems are faced by many children with cancer, and they can lead to negative physical and psychological outcomes, as well as to a lower quality of life. Educating families on healthy sleep hygiene to reduce sleep problems is important. We explored the use of a social robot to provide sleep hygiene education, as social robots seem to be an innovative and suitable tool for children. We developed an interactive education program during which the robot discussed six sleep hygiene topics with 28 children by asking them questions, followed by providing them information. We used multiple methods and found that the use of a social robot at the outpatient clinic was feasible, that children and parents had mostly positive experiences, and that the sleep hygiene of children was better two weeks after the education regimen. Our work may underline the value of providing education through a social robot. Further research is needed to develop and implement this intervention.

**Abstract:**

Objectives: Children with cancer often experience sleep problems, which are associated with many negative physical and psychological health outcomes, as well as with a lower quality of life. Therefore, interventions are strongly required to improve sleep in this population. We evaluated interactive education with respect to sleep hygiene with a social robot at a pediatric oncology outpatient clinic regarding the feasibility, experiences, and preliminary effectiveness. Methods: Researchers approached children (8 to 12 years old) who were receiving anticancer treatment and who were visiting the outpatient clinic with their parents during the two-week study period. The researchers completed observation forms regarding feasibility, and parents completed the Children’s Sleep Hygiene Scale before and two weeks after the educational regimen. The experiences of children and parents were evaluated in semi-structured interviews. We analyzed open answers by labeling each answer with a topic reflecting the content and collapsed these topics into categories. We used descriptive statistics to describe the feasibility and experiences, and a dependent-samples *t*-test to evaluate the preliminary effectiveness. Results: Twenty-eight families participated (58% response rate) and all interactions with the robot were completed. The children and parents reported that they learned something new (75% and 50%, respectively), that they wanted to learn from the robot more often (83% and 75%, respectively), and that they applied the sleeping tips from the robot afterwards at home (54%). Regarding the preliminary effectiveness, children showed a statistically significant improvement in their sleep hygiene (*p* = 0.047, *d* = 0.39). Conclusions: Providing an educational regimen on sleep hygiene in a novel, interactive way by using a social robot at the outpatient clinic seemed feasible, and the children and parents mostly exhibited positive reactions. We found preliminary evidence that the sleep hygiene of children with cancer improved.

## 1. Introduction

Sleep problems are common during and after treatment for childhood cancer [1,2,3,4] and they are related to a lower quality of life [5,6,7]. The prevalence of sleep problems in children with cancer during treatment ranges from 74 to 95% [5]. In this population, the sleep problems that are most frequently reported are bedtime resistance, sleep onset delay, and sleep anxiety. We can address these sleep problems with behavioral and educational interventions [1,6,8]. In the general population, parental knowledge about sleep hygiene is limited [9,10,11]. However, it has been shown that a greater parental knowledge of sleep is associated with healthier sleep practices [11,12] and that sleep hygiene education for parents and children is effective at improving sleep [13,14,15,16]. A multicomponent sleep intervention for children with brain tumors was tested in a pilot randomized controlled trial, which showed a modestly positive effect on nighttime sleep duration [17]. However, this intervention included education as well as relaxation training and stimulus control; thus, the effects of an education-only session on sleep hygiene in this population remains unknown. 

To effectively change sleep hygiene in school-aged children, education should not only target parents, but it should also positively engage children. Social robots are considered fun and motivational by children, and parents appreciate that robots have a lot of patience, are not judgmental, and can help their children [18]. Children also accept the instructions of social robots and enjoy the company [19]. In a recent review on social robots for education, it was found that robots can provide outcomes that are similar to human tutoring in specific tasks, and that they can improve cognitive and affective outcomes [20]. Social robots also showed promise in improving the knowledge of children in health care settings through the provision of information [21]. For instance, in a previous study, it was found that social robots can support health education for children with diabetes. When compared with a control group, the social robot group experienced more enjoyment and engagement, and there was an increase in their health knowledge [22]. 

Supporting families of children with cancer with an educational sleep hygiene intervention is important for improving their knowledge of sleep hygiene. A social robot could be an appropriate tool, but it has not been previously used for this purpose. Therefore, this study aimed to explore the use of a social robot for interactive sleep hygiene education among school-aged children with cancer and their parents. Specifically, we aimed to evaluate the feasibility, the experiences of the children and their parents, and the preliminary effectiveness on sleep hygiene. 

## 2. Methods

### 2.1. Participants and Recruitment

In this prospective study, children participated in an interactive sleep education session with a social robot together with one or both of their parents. Children were eligible to participate if they: (1) were between 8 and 12 years old, (2) had received active anti-cancer treatment, (3) visited the outpatient clinic of the Princess Máxima Center during the two-week study period, (4) were accompanied by at least one parent, and (5) were fluent in Dutch. Children did not have to experience sleep difficulties to participate. We selected the specific age range of 8 to 12 years for multiple reasons. We based the lower age limit on previous clinical experience with this specific robot, from which we concluded that children between 4 to 7 years old seemed too young for the type of interactions that were created. In addition, 8-year-old children are old enough to independently participate and go to bed autonomously. The upper age limit was set to prevent the inclusion of children with shifting circadian rhythms in adolescence [23,24], or the perception of the robot as too childish or patronizing.

We identified children who met all inclusion criteria and informed these families about the study by mail one week prior to their outpatient clinic appointment. Additionally, we contacted families by phone on the day before their visit to discuss any remaining questions regarding study participation, or to record reasons for nonparticipation. Parents of participating families signed informed consent. The institutional medical ethics review board (number 21/640) classified this study as exempt from the Medical Research Involving Human Subjects Act.

### 2.2. The Interactive Sleep Education 

**Figure 1:**
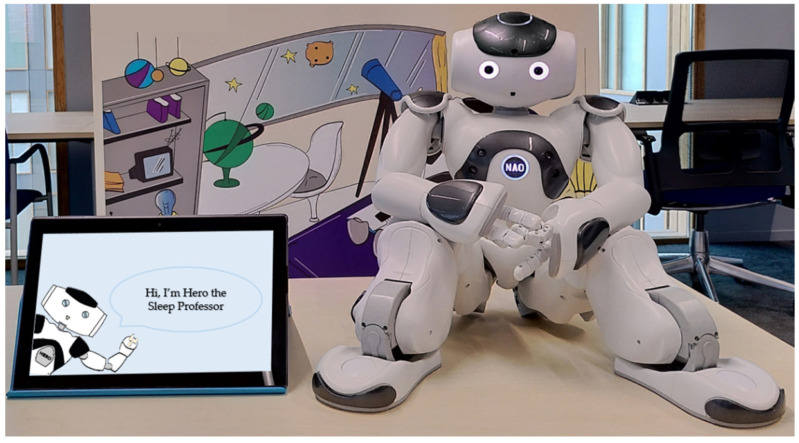
Picture of the robot and set-up.

For the interactive sleep education, we used the NAO^6^ robot (hardware produced by SoftBank Robotics ©). Figure 1 shows a picture of the robot and set-up. A software framework was developed to allow the robot to autonomously behave in a socially intelligent way. We selected six health behaviors that are important for healthy sleep hygiene to implement in the education session [25]: (1) minimal activities and screens before bedtime, (2) a consistent sleep routine, (3) an adequate sleep environment, (4) management strategies for worries, (5) daytime exercise, and (6) limiting food and drinks. We developed the content to be appropriate for children from 8 to 12 years old by using simple and appropriate language, providing visual support in the form of pictures, and fitting the content into a session with a duration of 10 min, at most (to ensure that children could sustain their attention).

**Figure 2:**
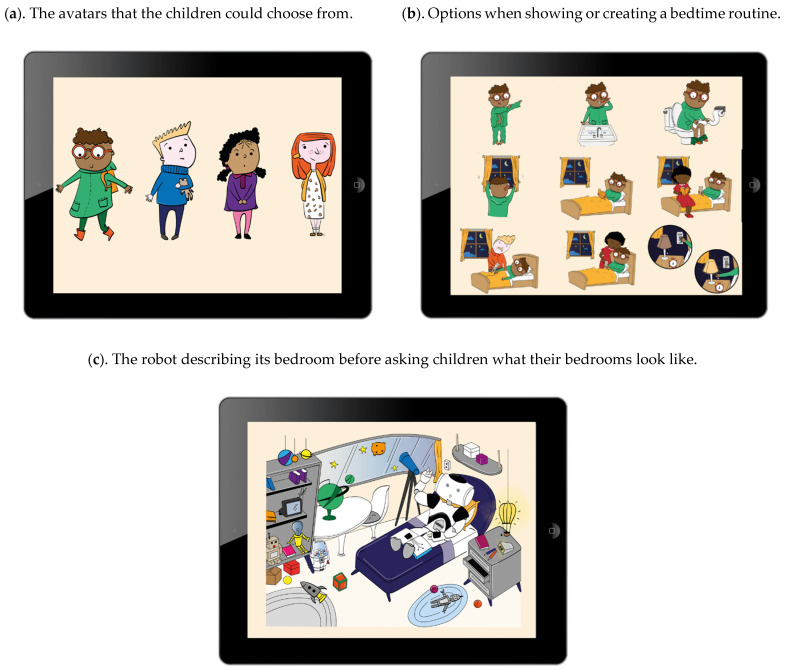
Examples of visual support by the tablet. © Illustrations by Patrizia D’Olivo.

The education started with the robot introducing itself as Hero the Sleep Professor, and with some small talk about sleep. The robot then discussed the sleep hygiene topics through 14 questions. The robot provided feedback on the children’s answers and delivered explanatory information. Whenever the robot provided a sleep hygiene tip, its lights turned orange to increase awareness. A tablet visually supported the information provided by the robot, and children were able to select a virtual avatar to represent themselves when performing exercises, such as creating a bedtime routine. Figure 2 shows the illustrations of the avatars and examples of the use of the tablet. At the end, the robot said goodbye, and then children received a magnet with a summary of the tips and a written relaxation exercise to take home. Table 1 shows more information regarding the content of the education and interactions.

The 14 questions were either generic (*n* = 8) or personalized (*n* = 6). For the generic questions, children could give any answer using speech or the tablet, and the robot would react the same regardless of the answer. For the personalized questions, children had to give specific answers, and the robot replied differently based on the answer. Here, children had two attempts to answer using speech. In case of repeated failures, the robot would use a repair mechanism by displaying multiple-choice options on the tablet to reduce frustration and to ensure that the interaction could properly continue [26]. 

We included two small breaks to allow children to relax and to support their attention span. During the first break, children recorded their own voices, which were used later in the education (i.e., co-creation) [27]. During the second break, children were invited to perform a popular TikTok dance together with the robot to music. The children could respond to the break activities by either participating or not, and the education continued regardless of their responses. 

### 2.3. Procedure and Measures

In the assessment of feasibility, researchers asked eligible patients whether it was possible for them to participate, as well as their reasons for wanting to participate. If they participated, then parents completed a questionnaire on their child’s sleep hygiene before the education session. One of the five trained research staff members guided the education session. To further assess feasibility, researchers completed an observation form to log the technical functioning of the robot, engagement of the child, and the course of the interaction between the child and the robot during the session. After the education session, researchers conducted semi-structured interviews with children and parents about their experiences with the robot. The interviews consisted of open and closed questions using an overall rating on a scale of 0 to 10 (with a higher score indicating a better experience). Two weeks after the interaction, parents completed the sleep hygiene questionnaire again, with two additional questions to evaluate the use of the tips. The total time investment for families was about 30 min. 

Sleep hygiene was assessed with the Dutch version of the Children’s Sleep Hygiene Scale (CSHS) [28,29]. This parent-report questionnaire consists of 25 questions about sleep hygiene that can be answered on a 6-point scale (1 = never and 6 = always). The CSHS provides an overall measure of sleep hygiene, where higher scores indicate better sleep hygiene. Reliability of the Dutch version is acceptable, with a Cronbach’s alpha of 0.78 [30]. 

### 2.4. Data Analyses

Data was analyzed using IBM SPSS Statistics, version 25. We used descriptive statistics (frequencies, percentages, and averages) to describe the sample, feasibility (possibility of and reasons for participating, technical functioning of the robot, engagement of children, and course of the interactions), and the experiences of children and parents. For the latter, we analyzed the open answers of the semi-structured interviews by labeling each answer with a topic reflecting the content. Two researchers (KvB and HvdH) independently identified and coded the topics of the open answers and collapsed them into categories. The researchers discussed the differences until they reached a consensus. To determine preliminary effectiveness of the program on sleep hygiene, a total mean score was calculated for the CSHS. We used a repeated measures *t*-test (*p* < 0.05) to analyze differences in sleep hygiene scores before and two weeks after the education. We estimated Cohen’s *d* to interpret the magnitude of the effect, where we considered 0.2 as small, 0.5 as medium, and >0.8 as large, based on Cohen’s guidelines [31].

## 3. Results

### 3.1. Participants

The participants (*n* = 28) were 9.4 years old (SD = 0.99), on average, and they were evenly divided regarding sex (50% boys). Most children were diagnosed with a hemato-oncological disease. The parents of eight children (29%) shared additional information about their children: they reported two cases of Down syndrome, a developmental delay, a visual impairment, Gilles de la Tourette, speaking and performance anxiety, selective mutism, and autism with ADHD. Table 2 shows more details about the participants’ characteristics. 

### 3.2. Feasibility

#### 3.2.1. Possibility of Participating When Visiting the Outpatient Clinic

**Figure 3:**
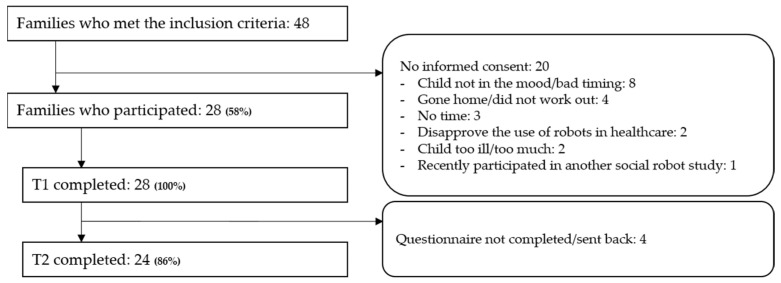
Flowchart of participants and reasons for not participating. Note: T1—interactive sleep hygiene education at the outpatient clinic; T2—completing sleep hygiene questionnaire two weeks later.

A total of 48 families met the inclusion criteria and were invited to participate in the study. There were 28 families that participated (58%). There were 20 families that did not participate, which was mostly because the children were not in the mood/it was not a good moment (42%). Of the 28 families that participated, 24 families (86%) also completed the questionnaire two weeks later. Figure 3 shows more details about the inclusion process and reasons for not participating.

#### 3.2.2. Reasons for Wanting to Participate

Children reported that they participated because they were interested in the robot (61%), but also because their parents wanted them to (32%), or they wanted to learn more about sleep (7%). The reasons that the parents participated were more diverse: being interested in the robot (29%), helping researchers and science (29%), because their children wanted to (21%), to learn about sleep (18%), and to pass time at the hospital (4%).

#### 3.2.3. Technical Functioning of the Robot

The robot functioned without any problems in almost all the cases (89%). In the three cases where the robot did not function properly, restarting the robot solved the problem. For all the children (100%), the interaction with the robot could be fully completed.

#### 3.2.4. Participation of Children 

Almost all the children (93%) were involved in the interaction with the robot, and they maintained their engagement from beginning to end. Two children sometimes lost their attention span, but they still completed the interaction.

#### 3.2.5. Course of the Interaction between Child and Robot

Most of the children (89%) responded to all of the generic questions; two children did not respond one time, and one child did not respond twice. Children needed 1.5 attempts, on average, to provide an answer to a personalized question that the robot could understand and process. More than half of the children (54%) needed to answer using the repair mechanism on the tablet: eight children once, six children twice, and one child three times. The researcher often helped the children (50% with generic questions and 72% with personalized questions) during the interaction, mostly by providing extra instructions. The children (59%) needed the most help with the first two questions, and barely any help (3%) with the last two questions. At the breaks, most children (71%) participated in all of the activities. The children responded least to the robot asking them to dance and snore. 

### 3.3. Experiences of Children and Parents

#### 3.3.1. Evaluation by Children

**Figure 4:**
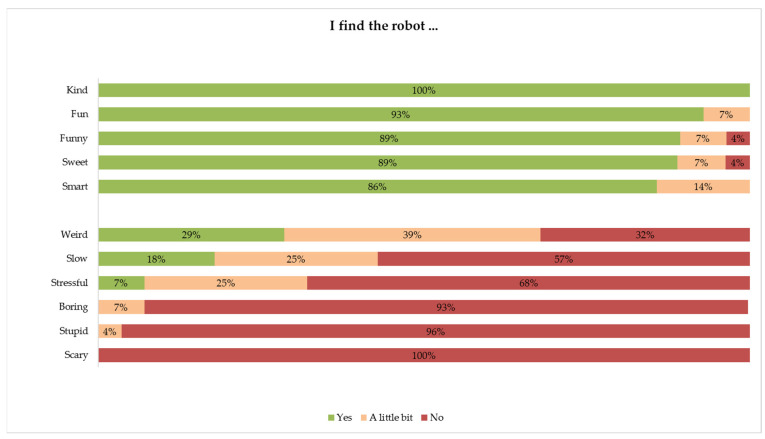
Ratings of the robot by children.

The children were generally enthusiastic about their interactions with the robot. The majority (55%) did not dislike any aspect of the robot, and none thought that the robot was scary. The children mentioned the interactive elements, such as talking and dancing with the robot, as the parts that they liked as well as disliked. Most of the children (75%) indicated that they learned something new about sleep from the robot, mostly regarding food and drinks and the sleeping routine. Furthermore, most of the children (75%) indicated that they intended to follow up on the sleeping tips of the robot, mostly with regard to limiting screen time before bed, paying attention to food and drinks, and sleeping in a dark room. Most of the children (82%) wanted to engage in interactive education with the robot more often. On average, the children rated their interaction as 8.6 (range: 5 to 10). Figure 4 shows more details about how the children evaluated the robot. 

#### 3.3.2. Evaluation by Parents

Almost all of the parents (96%) were positive about the use of a robot for educational purposes at the hospital, and most of the parents (86%) found the education appropriate for their children. The following are what parents considered the positive aspects: suitability for children, a playful way of learning, and the interactive nature of the robot. Most of the parents (71%) thought that their child learned something new from the education, and half of the parents (50%) learned something new themselves. Many of the parents did not have any suggestions for improvements (52%); however, if they did, they consisted of the following areas of inquiry: appropriateness for other ages (younger or older), presentation of the robot (location and looks), speaking pace (both faster and slower), and further software development (a better understanding of answers). Most of the parents (75%) indicated that they would want to engage in interactive education with the robot more often. In addition, parents suggested other topics for this: medical procedures; pain, stress, and anxiety; nutrition; and medication. They suggested that the robot could be helpful for distraction, entertainment, physical activity, and to accompany the children as a buddy. On average, the parents rated the interaction as 8.0 (range: 7 to 10).

### 3.4. Preliminary Effectiveness on Sleep Hygiene

Two weeks after the sleep education session with the robot, about half of the parents (54%) reported to have implemented something from the education at home, which mostly included rules to limit screens and stimulus control before bed. The parents who did not implement elements from the education indicated that they already applied most of the tips. Before the education, the sleep hygiene scores of the children ranged from 4.40 to 5.56 (*M* = 5.11, SD = 0.27). Two weeks after the education, the sleep hygiene scores ranged from 4.76 to 5.68 (*M* = 5.26, SD = 0.25), which was a statistically significant improvement (*M*_diff_ = 0.10, *t*(22) = −2.1, *p* = 0.047), with a small to medium effect size (*d* = 0.39). 

## 4. Discussion

In the current study, we investigated interactive education with respect to sleep hygiene with a social robot at a pediatric oncology outpatient clinic. The execution of the education at the outpatient clinic was feasible, as more than half of the families were able to attend, and the interest in participation was high. Moreover, the setting of the education session with the social robot was inclusive, as several children with (developmental) comorbidities were able to participate and complete the educational session together with their parents. The robot functioned well, and all the children were able to complete the educational regimen. The repair mechanism and the support of the tablet were important factors that contributed to the feasibility of the education session. The children responded well to the interactive parts, and they were able to maintain their attention for the 10-min session. Even though they often needed help from the researcher, the children quickly learned (in minutes) how to interact with the robot as their education progressed, which is similar to another study in healthy children [32].

The children and parents reported positive experiences regarding the education session, which is comparable to other social robot studies [18,33,34]. Contrary to previous research, the children did not remark on the repetitive aspects of the education regimen and the slow responses of the robot [22]; however, we had a one-session interaction, compared with the multiple-session education of the other study. Considering that families reported that they would like to learn from the robot more often, multiple sessions seem appropriate and could be beneficial for educational purposes [22,35].

The children enjoyed the interactive parts of the educational session, but some of the children disliked the interactive activities during the breaks. This result was also found in another study with healthy children who engaged in interactive storytelling with a social robot [36]. A minority of the children disliked the interactive parts, and in a follow-up study, the researchers made parts of the interaction optional, which resulted in an improved sense of agency and acceptance [27]. Nevertheless, most of the families indicated that they learned something new from the robot, which we expected based on the literature [20,21,22], and this confirms that these results also apply to our pediatric outpatient oncology setting and for the purpose of sleep education.

The preliminary effectiveness of sleep education from a social robot in the outpatient pediatric oncology care setting was promising, as we found a statistically significant improvement in sleep hygiene two weeks after the interactive educational session. These results are remarkable, as one review concluded that while most sleep education programs in healthy children increased their sleep knowledge, this did not necessarily equate to sleep behavior changes, such as improved sleep hygiene [37]. However, in this study, most of the families followed up on the sleeping tips from the robot and applied the tips at home, such as less screen time and more relaxing activities before bedtime. This result may underline the additional value of including parents in the educational session and providing education through a social robot. The magnet with a summary of the sleep hygiene tips may have contributed to this effect as well. 

### 4.1. Clinical Implications

Most children with cancer experience sleep problems due to treatment effects, treatment-related toxicities, the hospital environment, and psychological and social factors [38,39,40,41]. These sleep problems are associated with many negative physical and psychological health outcomes [42,43], and consequently with a lower quality of life [6]. Therefore, interventions are paramount for improving sleep in this population [44], which may be achieved by improved sleep hygiene [14,16]. Our study achieved positive results in terms of feasibility, the experiences of the children and parents, and the indications of the program’s effectiveness, demonstrating that administering sleep hygiene education to children and parents via a social robot is a promising form of intervention for sleep problems.

### 4.2. Future Directions

A larger study of the program’s effectiveness is needed before a social robot that provides sleep education can be considered effective at improving the sleep hygiene of children with cancer. We evaluated the effects of one education session shortly after the intervention, and it is unknown whether these results will last over the long term, or whether repeated interactions would be meaningful for better or longer-lasting behavioral change. Future developments should focus on how to best design such interactions with social robots in a way that keeps children optimally engaged [45,46], and by taking into account the role of novelty [47]. In addition, researchers should investigate whether the intervention is specifically effective for children who experience sleep difficulties, as they are in the most need of support. 

Upon further implementation of a social robot in a pediatric oncology setting, it would be interesting to consider whether the educational session should be supervised. On the one hand, the presence of a researcher during the educational session was essential to provide guidance and address difficulties, but it was also a potential barrier. However, previous research on health care providers in pediatric oncology worldwide has shown that the majority of them would be open to using a social robot in their work [48]. On the other hand, social robots that can be independently used by families (for example, in the waiting room at the outpatient clinic) could be appealing as well and could provide opportunities for easily accessible (and fun) information and prevention, with a minimal burden on the health care staff.

### 4.3. Limitations 

During the interactions with the robot, the researcher sometimes helped the children. This was mainly based on the researcher’s judgement of its necessity, which may warrant caution when interpreting these results. Regarding our measures on sleep, asking the children whether they adopted any changes based on the educational session with the robot would have been a meaningful addition, as we only asked the parents. 

## 5. Conclusions

In this study, it was feasible to provide education on sleep hygiene in an interactive and playful way through a social robot at an outpatient clinic. The children undergoing cancer treatment and their parents were mostly positive about their experiences, and according to the preliminary results, the educational session had positive effects on the sleep hygiene of the children. Therefore, social robots appear to be a promising tool for education on sleep hygiene in pediatric oncology groups.

## Figures and Tables

**Table 1:** Components of the interactive sleep education.

Topics and Questions	Type of Interaction		
	Generic questions (*n* = 8)	Personalized questions (*n* = 6)	Activity (*n* = 4)
**Introduction and general talk about sleep**			
Hello, my name is Hero. What’s your name?	Speech (any)		
Can you pick a character you like or that looks most like you?	Tablet		
How do you sleep?	Tablet		
**1. Activities and screens before bedtime**			
What do you do before you go to bed?	Speech (any)		
**2. Consistent sleep routine**			
Do you have a consistent sleep routine before you go to sleep?		Speech (y/n/s)	
Do you want to show me your sleep routine using the tablet?/ Shall we make a sleep routine together on the tablet?		Speech (y/n)	
Can you put the images in the order of your own sleep routine?/ Can you put the images in an order that seems convenient to you?	Tablet		
**Break 1: co-creating**			
Could you applaud me? Just do in 3, 2, 1.			Participate
Could you cheer, as if you’ve just won a game? Just do in 3, 2, 1.			Participate
Could you let me hear how you snore? Just do in 3, 2, 1.			Participate
**3. Sleep environment**			
What does your bedroom look like?	Speech (any)		
Is your room dark when you go to sleep?	Tablet		
**4. Managing worries**			
When you go to bed, do you fall asleep easily?		Speech (y/n/s)	
I sometimes have trouble falling asleep because I’m worried, and I have to think about it all the time. Do you ever experience that?		Speech (y/n/s)	
**Break 2: dancing**			
Shall we dance for a moment?			Participate
**5. Daytime exercise**			
Exercise, like sports, playing outside or riding your bike can help you to sleep better. Do you exercise often?		Speech (y/n/s)	
**6. Food and drinks**			
I’m curious what food and drinks you think you shouldn’t take before you go to bed. Just click on them on the tablet.	Tablet		
**Goodbye**			
Did you enjoy our conversation as well?		Speech (y/n)	

Note: y/n/s: yes/no/sometimes.

**Table 2:** Participant characteristics (*n* = 28).

	*n*	%
Age		
8 years	7	25.0
9 years	6	21.4
10 years	12	42.9
11 years	3	10.7
Sex		
Boys	14	50.0
Girls	14	50.0
Diagnosis type		
Hemato-oncology	19	67.9
Neuro-oncology	7	25.0
Solid tumor	2	7.1

## Data Availability

The data presented in this study are available upon request from the corresponding author. The data are not publicly available due to privacy restrictions.
